# Delayed Presentation of Penetrating Abdominal Trauma From a Nail Gun: A Case Report

**DOI:** 10.7759/cureus.82456

**Published:** 2025-04-17

**Authors:** Aws E Ahmed, Ricardo J Lopez Hanson, Keelin F Roche, Bracken Burns

**Affiliations:** 1 Department of Surgery, East Tennessee State University Quillen College of Medicine, Johnson City, USA

**Keywords:** accidental trauma, general trauma surgery, major trauma, nail gun injury, penetrating abdominal injury

## Abstract

Penetrating abdominal trauma typically presents in an acute manner, and most require emergent intervention due to small bowel, large bowel, and liver injury. Here, we report a 53-year-old man who sustained multiple penetrating intra-abdominal injuries from a nail gun. The patient did not seek medical attention until four weeks after the incident. He presented with worsening abdominal pain and underwent exploratory laparotomy and removal of three intact nails. Fortunately, there were no injuries to the intra-abdominal organs, and the patient had an uneventful post-operative course and was seen in the office after hospital discharge in good condition.

## Introduction

Nail gun penetrating injuries are not uncommon [1]. The majority of nail-gun-related injuries involve the extremities [2,3]; however, injuries to almost every part of the body have been described [4]. Like most penetrating abdominal trauma, penetrating injuries from a nail gun typically follow an acute presentation [5]. Although nail gun injuries to the abdomen are rare compared to stab wounds and gunshot wounds, they pose a challenging case due to the small entry wound and high-velocity impact that can damage critical structures. The majority of nail gun traumas are accidental due to work-related injuries in construction or home improvement projects [6]. This case report describes the unusual, delayed presentation of a 53-year-old man who sustained multiple penetrating intra-abdominal injuries from a nail gun. 

## Case presentation

A 53-year-old male presented to an outside hospital with complaints of abdominal discomfort. He has a previous history including proctocolectomy with end ileostomy for ulcerative colitis, not on steroids or biologic medications for several years. About four weeks before his presentation, he used a pneumatic nail gun to fasten wood pieces braced against his abdomen. He had recently bought a new nail gun and was still working on adjusting the calibration. He did not explicitly recall shooting himself in the abdomen, but he did notice three small wounds on his abdominal wall after the incident. He denied ever having abdominal pain, fevers, chills, or a change in ostomy output.

**Figure 1:**
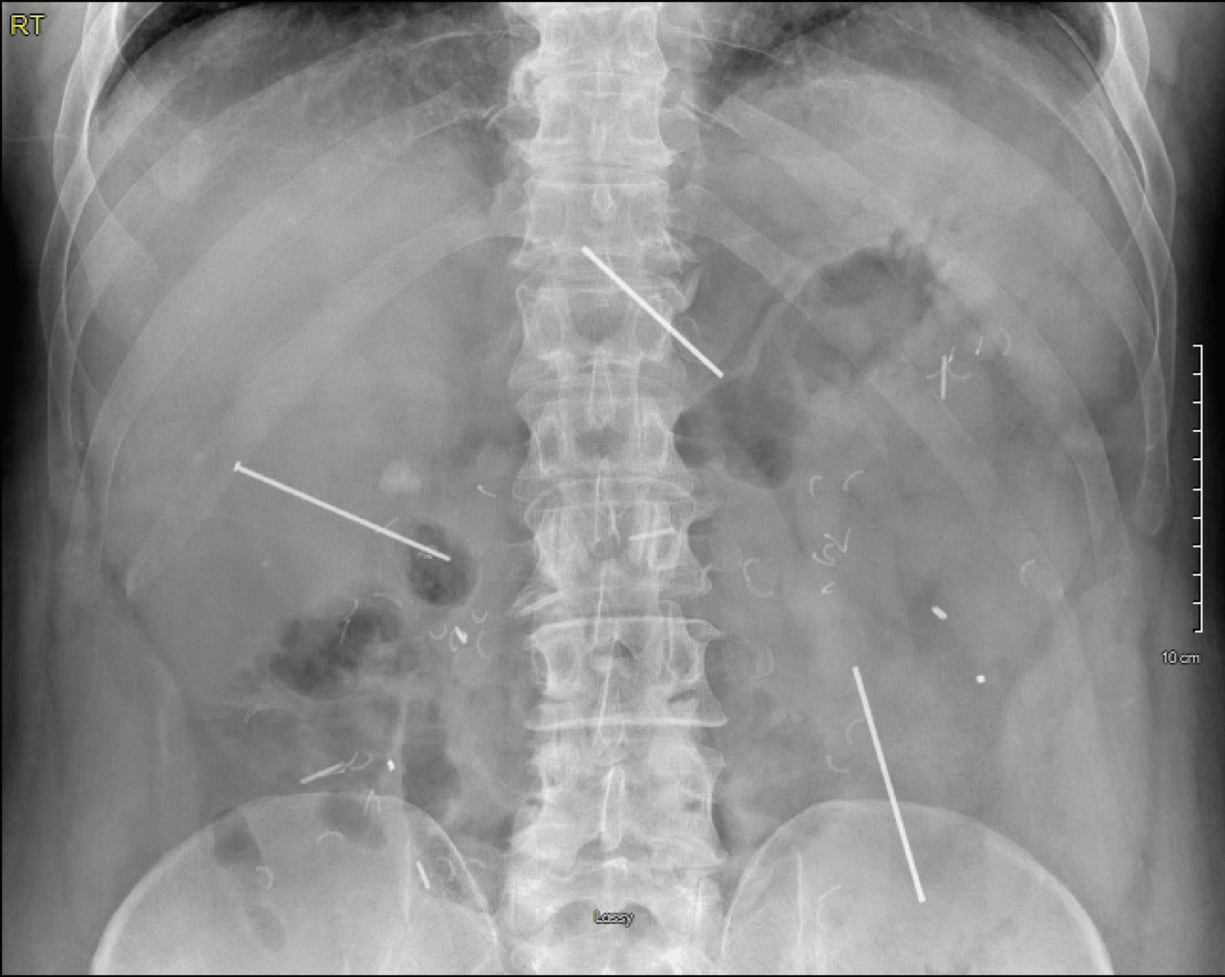
Three nails within the abdominal cavity on abdominal X-ray

**Figure 2:**
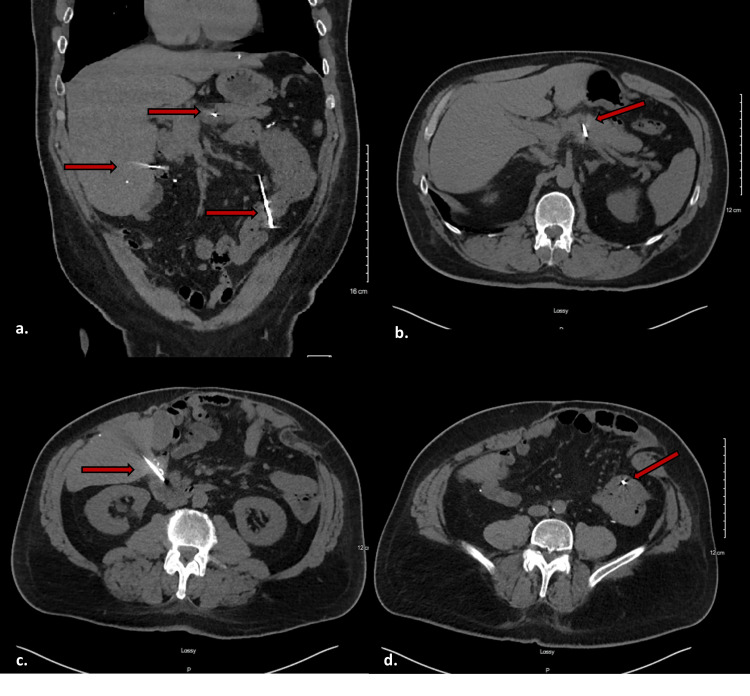
CT scan of the abdomen and pelvis showing nail locations a) Three nails seen within the abdominal cavity (red arrows); b) Nail traversing the left lobe of the liver and the middle of pancreas (red arrow). c) Nail traversing the right lobe of the liver and abutting the duodenum (red arrow). d) Nail either within or between the small bowel and mesentery (red arrow).

Several weeks later, he developed vague abdominal discomfort for which he was seen in an urgent clinic and was discharged due to a benign abdominal exam, which was thought to be due to an ulcerative colitis flare. As his abdominal pain persisted, he presented to the emergency department. An abdominal X-ray demonstrated three linear foreign bodies (Figure 1). Follow-up CT imaging demonstrated what appeared to be three intact nails within the abdomen. One projected through the left lobe of the liver and through the mid-pancreas, with the tip adjacent to the splenic artery and vein. The second projected through the inferior right lobe of the liver and ended either within or adjacent to the second portion of the duodenum. The third appeared to be within the left abdominal mesentery either through or adjacent to loops of small bowel (Figure 2).

Despite the radiographic findings, the patient did not have peritonitis, and vital signs were all stable without fever or significant laboratory abnormalities. Given the risk of delayed migration that may cause bowel obstruction vs perforation, the patient elected to proceed to the operating room for a laparotomy to define the extent of the injury and retrieve the foreign bodies. A standard midline laparotomy was performed, and the intra-abdominal cavity was inspected for foreign bodies. A laparoscopic approach was not considered, given the patient's previous abdominal surgery and the locations of the nails.

**Figure 3:**
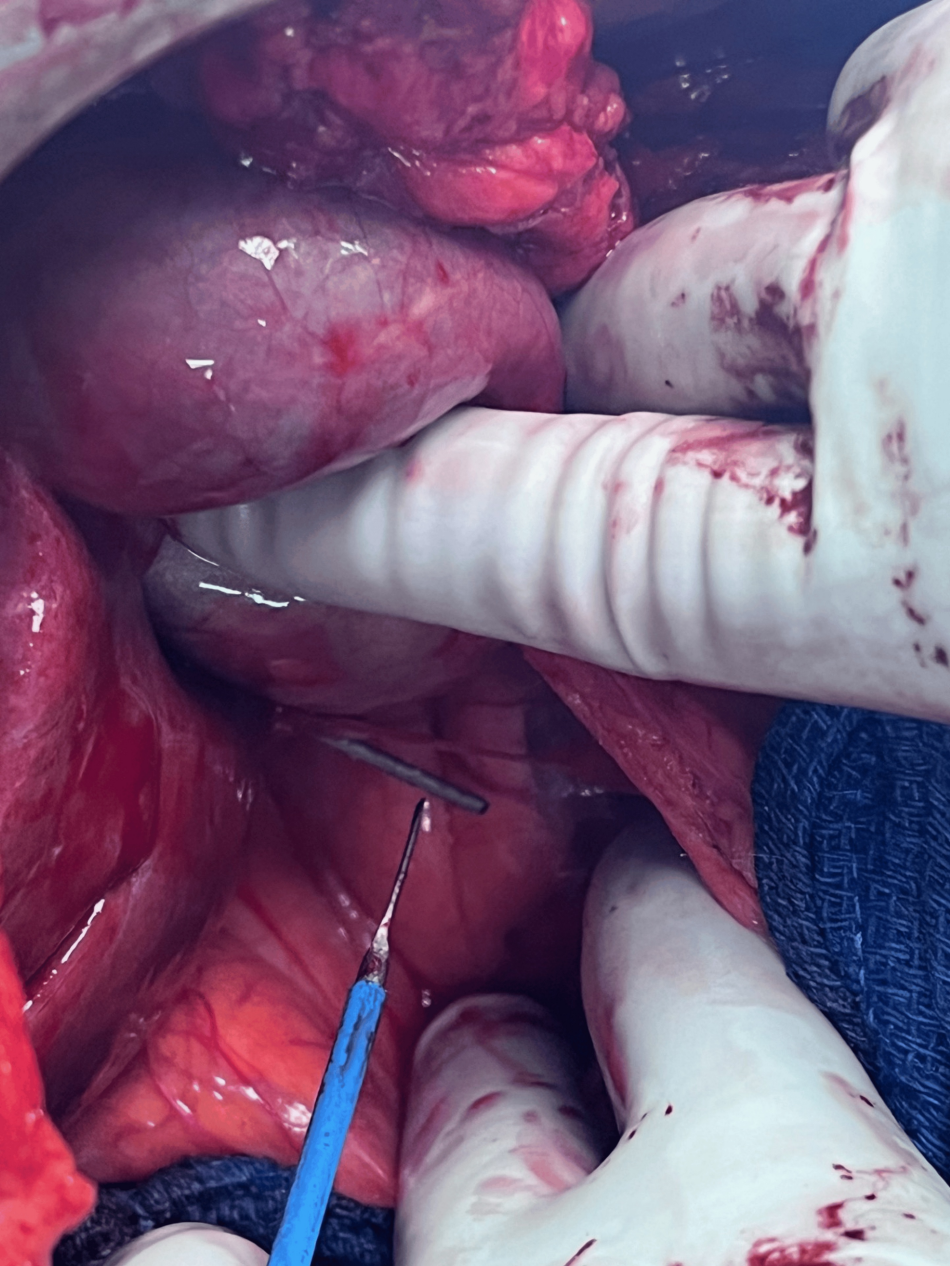
Intraoperative photo of the second nail The second nail that appeared to have traversed the right lobe of the liver but stopped just short of the duodenum

**Figure 4:**
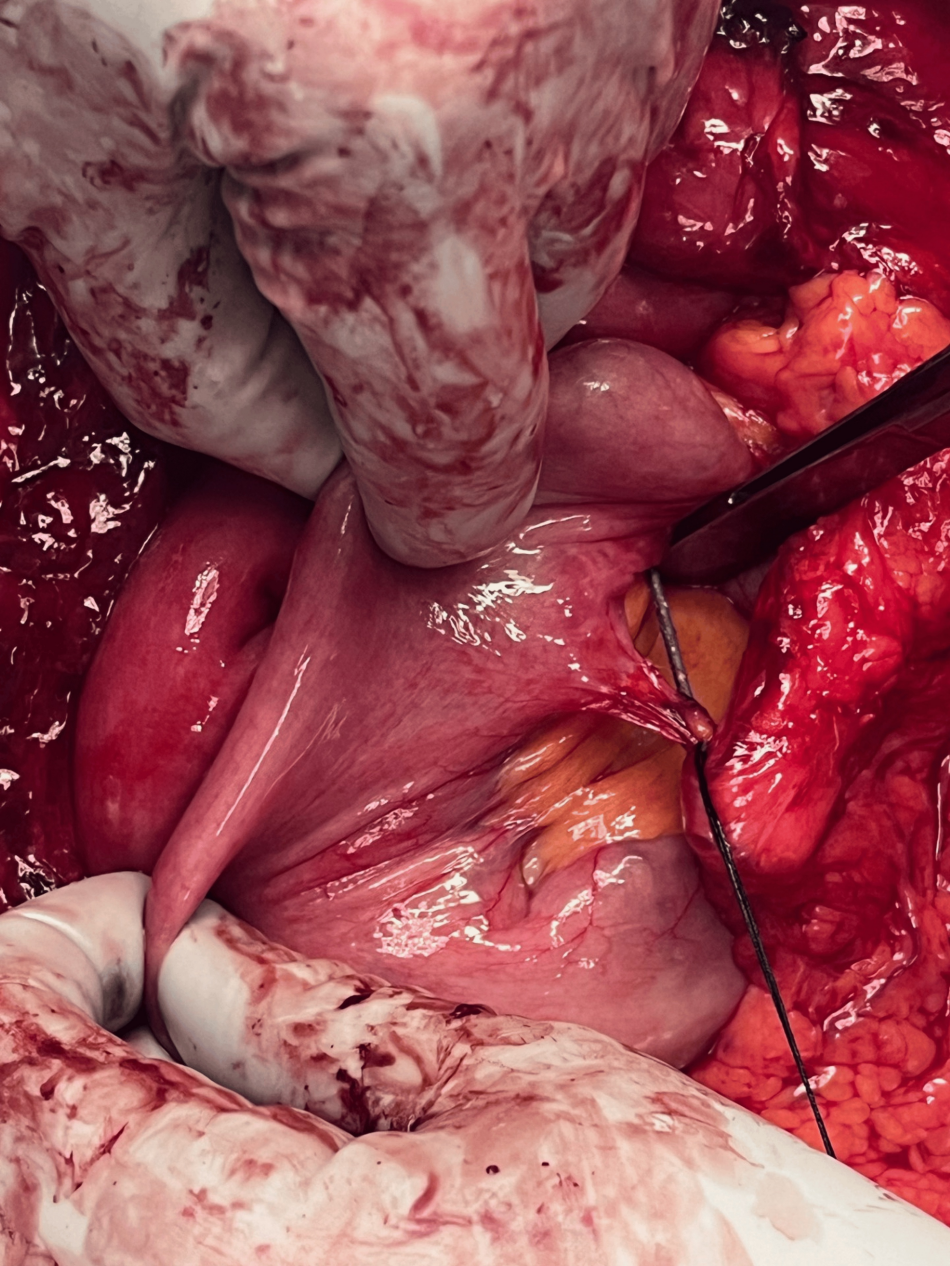
Intraoperative photo of the third nail The third nail was through the small bowel mesentery in the left abdomen and rested between loops of small bowel

**Figure 5:**
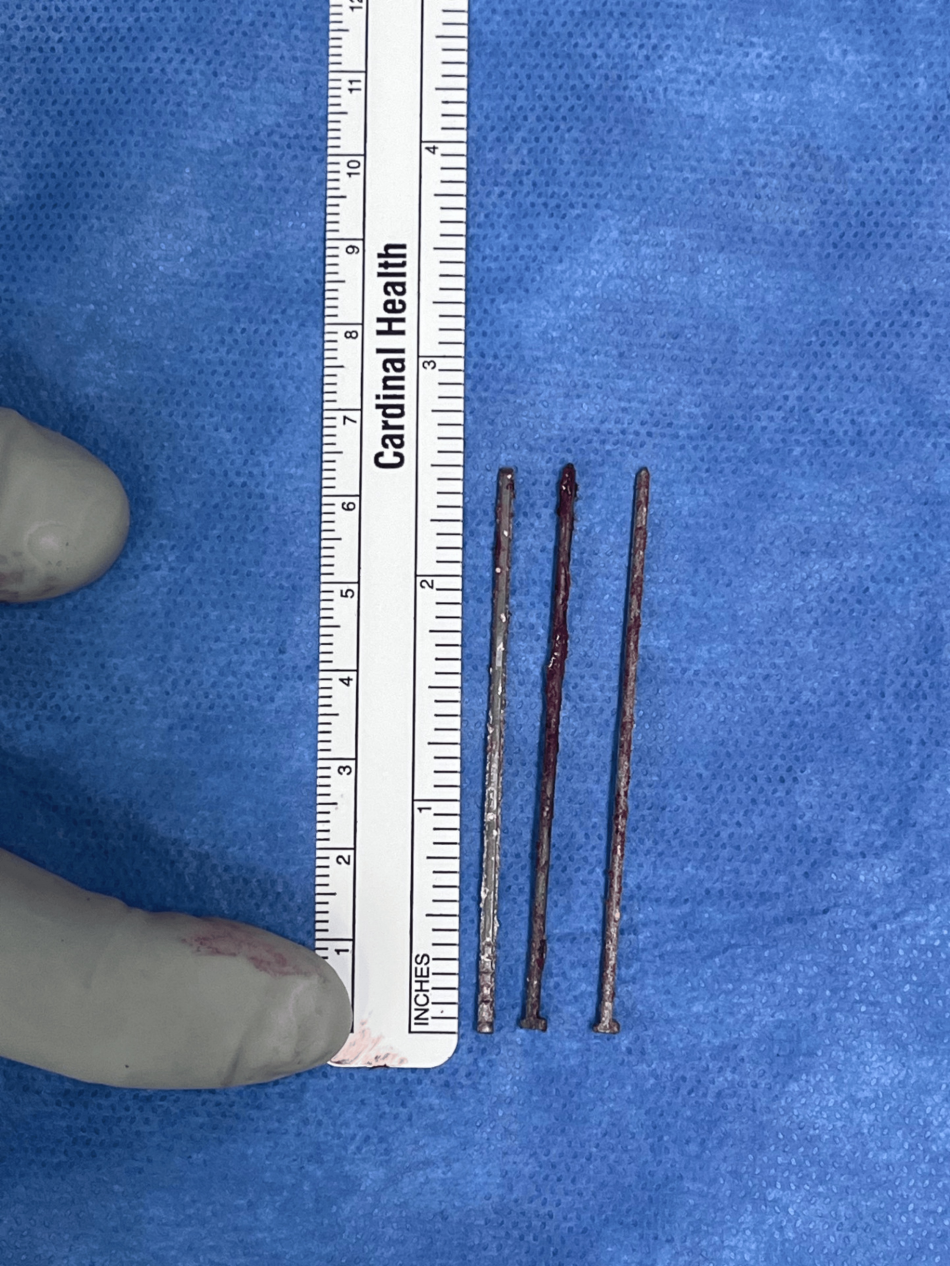
Nails after extraction

The first nail was identified; it traversed through the left lobe of the liver and the mid-pancreas. Once removed, there was no leakage of bile from the liver, and no pancreatic fluid was observed. The splenic vasculature was not involved. The second nail that ended near the duodenum appeared to have traversed the right lobe of the liver but stopped just short of the duodenum (Figure 3). The tip of the nail abutted the lateral wall of the second portion of the duodenum without penetration. The third nail was through the small bowel mesentery in the left abdomen and rested between loops of the small bowel (Figure 4). No bowel injury was found. All three nails were removed without incident (Figure 5). The midline fascia was closed primarily. Drains were left alongside the pancreas and the duodenum. The postoperative course was uneventful, and the drains were removed during his hospital stay. The patient reported that the abdominal pain that prompted his presentation was resolved, and the patient was seen two weeks postoperatively in the office in good condition without any abdominal pain or complication, his midline staples were removed. The patient was advised to follow up in the surgery clinic in six months for monitoring of any long-term complications.

## Discussion

There are an estimated 25,000 cases related to nail gun injuries reported every year [1]. While most cases involving penetrating trauma follow an acute presentation requiring urgent surgical intervention [5], delayed presentation or late manifestation of injury is possible depending upon the mechanism of the trauma and anatomical location. Other patient factors can influence the presentation, such as immunosuppression.

The majority of nail gun injuries are accidental discharge that happens at construction sites or during home improvement activities, although suicide attempts using these devices have been reported [6]. Nail guns are considered high-velocity devices that can eject nails as fast as 1400 feet per second [2]. Given the small entry sites associated with these nails, a thorough physical exam should be performed, and in case of abdominal entry, ruling out peritoneal signs such as rigidity, guarding, and rebound tenderness is important, as this warrants urgent surgical exploration. Plain X-rays can detect nails given their radiopaque nature, but a computed tomography scan is the gold standard for evaluating these injuries as it will identify vascular injuries, organ laceration, and bowel perforation.

Treatment of pneumatic nail injuries usually varies based on anatomical location and patient presentation. In cases of hemodynamic instability, such as peritoneal signs or vascular injuries, surgical intervention is warranted acutely. For patients without hemodynamic instability and isolated extraperitoneal injuries, observation and non-operative management can be considered [7].

We discussed the risks and benefits of retrieving the nails in this case. Apart from mild abdominal discomfort, the patient was not acutely ill and did not exhibit signs of peritonitis. However, given the progression of his pain, we felt that retrieval of the nails and thorough exploration to define the extent of injury was warranted. Case reports describing delayed biliary obstruction from the migration of retained ballistic objects over time are rare [8,9]. For example, Rescorla et al. [10] demonstrated biliary obstruction from the migration of a bullet to the left hepatic duct 22 months following the initial presentation of a gunshot wound to the right upper quadrant. On initial exploration, there were no injuries identified to the hepatic duct.

We discussed with the patient the risk of bleeding, bile, pancreatic, or duodenal leak, and possible relocation of his ileostomy. We felt that leaving the nails carried a risk of possible future erosion into the bowel, biliary, or pancreatic duct system, as previously reported.

This case's significance is highlighted by the delayed presentation after multiple big caliber nail gun injuries into the abdominal cavity without organ damage or complication. This is highly unusual for penetrating abdominal trauma, which usually follows acute presentation and requires emergent intervention [5]. This case is also unique given the patient's previous history of inflammatory bowel disease and delayed awareness of possible foreign bodies until he presented to the hospital four weeks later with worsening abdominal pain. It should be emphasized that even with the absence of hemodynamic instability and peritonitis on exam, operative exploration should still be considered to avoid the risk of delayed complications. 

## Conclusions

Most traumatic pneumatic nail gun injuries usually involve the extremities; this case illustrates a rare presentation of delayed trauma secondary to an unintentional pneumatic nail gun injury to the abdomen. Upon operative exploration, there were three intact nails identified in the intra-abdominal cavity traversing critical structures, including the liver, pancreas, and small bowel mesentery, without significant injury. Surgical intervention with laparotomy retrieved all three nails with no significant organ damage or delayed perforation, with uneventful postoperative recovery and resolution of abdominal pain. This case highlights the atypical pattern of these retained foreign bodies, with rare cases reported causing delayed perforation or migration. Nail guns, while seeming to be less dangerous than firearms, are still capable of causing significant injuries, given their high velocity and ability to penetrate crucial organs and even bones. It is important to follow safety protocols, have proper training, and develop safety features to prevent serious work-related injuries when using these tools.
